# Predictors Of Cognitive Intra-Individual Variability In Aging Adults With HIV From The Deep South

**DOI:** 10.1093/geroni/igaf122.2680

**Published:** 2025-12-31

**Authors:** Xueling Zeng, Hathaichanok Phaowiriya, Maryam Rostamvand, Pariya Fazeli, Raymond Jones, Frank Puga, Elizabeth Byrd, David Vance

**Affiliations:** The University of Alabama at Birmingham, Birmingham, Alabama, United States; The University of Alabama at Birmingham, Birmingham, Alabama, United States; The University of Alabama at Birmingham, Birmingham, Alabama, United States; University of Alabama at Birmingham, Birmingham, Alabama, United States; The University of Alabama at Birmingham, Birmingham, Alabama, United States; University of Alabama at Birmingham, Birmingham, Alabama, United States; The University of Alabama at Birmingham, Birmingham, Alabama, United States; University of Alabama at Birmingham, Birmingham, Alabama, United States

## Abstract

While conventional cognitive assessments focus on capturing mean-based performance, they often overlook naturally occurring fluctuations in cognitive performance across cognitive domains (i.e., executive functioning). Referred to as cognitive intra-individual variability (IIV), it is associated with poorer cognitive and everyday functioning, can predict cognitive impairments, and is associated with morbidity and mortality. Yet, few studies have examined cognitive IIV in aging people with HIV (PWH). Our study examined factors associated with cognitive IIV in 260 PWH aged 40+ who were administered a comprehensive cognitive and psychosocial battery. For this analysis, we used cross-sectional data to explore the correlations among demographic, HIV characteristics (e.g., CD4+ count), medication adherence, psychosocial factors (depression, personality traits, locus of control), and cognitive IIV. Cognitive IIV (iSD) was measured through the variation across cognitive domains. In this sample from Birmingham, Alabama, most were male (63.85%) and African American (82.70%), with a mean age of 51.15 years. Results showed that higher IIV was positively correlated with locus of control (r = 0.26), suggesting that individuals who believe outcomes are out of their control lead to less stable cognitive performance. Higher scores on openness (r = 0.27) and agreeableness (r = 0.21) were associated with lower cognitive IIV. Surprisingly, no single cognitive domain predicted cognitive IIV (r’s=-0.14 to -0.22). Although exploratory, these findings suggest that cognitive IIV may have a subtle psychosocial profile, suggesting such cognitive fluctuations may have an impact of perception and behavior. Techniques to alter cognitive IIV as people age are being developed which could impact such perceptions are posited.

